# Diet-induced obesity impairs spermatogenesis: a potential role for autophagy

**DOI:** 10.1038/srep43475

**Published:** 2017-03-09

**Authors:** Yang Mu, Wen-jie Yan, Tai-lang Yin, Yan Zhang, Jie Li, Jing Yang

**Affiliations:** 1 Reproductive Medicine Center, Renmin Hospital of Wuhan University, Wuhan 430060, China; 2 Department of Obstetrics and Gynecology, Renmin Hospital of Wuhan University, Wuhan 430060, China

## Abstract

Autophagy is an evolutionarily conserved process that plays a crucial role in maintaining a series of cellular functions. It has been found that autophagy is closely involved in the physiological process of spermatogenesis and the regulation of sperm survival and motility. However, the role of autophagy in high-fat diet (HFD)-induced impaired spermatogenesis remains unknown. This study was designed to investigate the role of autophagy in HFD-induced spermatogenesis deficiency and employed chloroquine (CQ) to inhibit autophagy and rapamycin (RAP) to induce autophagy. 3-methyladenine (3-MA) and CQ were administered via intratesticular injection *in vivo*. The effects of CQ and 3-MA on the parameters of spermatozoa co-cultured with palmitic acid (PA) *in vitro* were also investigated. Human semen samples from obese, subfertile male patients were also collected to examine the level of autophagy. The results suggested that HFD mice subjected to CQ showed improved spermatogenesis. Inhibiting autophagy with CQ improved the decreased fertility of HFD male mice. Moreover, the *in vivo* and *in vitro* results indicated that both CQ and 3-MA could suppress the pathological changes in spermatozoa caused by HFD or PA treatment. Additionally, the excessive activation of autophagy was also observed in sperm samples from obese, subfertile male patients.

Infertility is defined as the failure to achieve a clinical pregnancy after 12 months or more of regular unprotected sexual intercourse^1^,^2^. Approximately 25–30% of the couples experiencing infertility have male-factor infertility^3^. Obesity is an acknowledged risk factor for male subfertility^4^,^5^,^6^,^7^. The mechanisms underlying obesity-induced male spermatogenesis deficiency remain unclear; deciphering these molecular mechanisms could be of great therapeutic interest for spermatogenesis impairment.

The mechanisms underlying spermatogenesis impairment induced by obesity are complex. Endocrine disorders^8^,^9^,^10^,^11^, genetic components^12^,^13^,^14^,^15^ and physical or chemical factors^16^,^17^ are all involved in the development of subfertility caused by obesity. However, accumulating data indicate a potential role of autophagy in spermatogenesis^18^,^19^.

Autophagy, or programmed cell death type II, is an evolutionarily conserved process that plays a crucial role in maintaining a series of physiological functions through the formation of a double-membrane vesicle termed the autophagosome followed by subsequent fusion with lysosomes and degradation of the cytosolic components by resident hydrolases^20^,^21^. Autophagy activates apoptosis, which could lead to oligozoospermia and infertility^22^,^23^,^24^. Autophagy also regulates inflammation^25^, which is closely associated with male spermatogenesis impairment^26^,^27^,^28^,^29^. Moreover, a recent study indicated that autophagy plays a key role in the impairment of spermatogenesis after heat treatment^30^. Germ cell-specific knockout of autophagy-related gene 7 (*Atg7*) causes male infertility in mice^19^. Autophagy was also indicated to be involved in the regulation of sperm survival and motility^18^. Autophagy was found to be activated under specific circumstances such as nutrient depletion, growth factor depletion and hypoxia^31^,^32^. Previous study indicated that mice fed with high-fat diet (HFD) had reduced hepatic autophagy^33^, while others reported that nondiabetic HFD mice had upregulated autophagosome formation^34^. In addition, autophagy is associated with many HFD-related diseases such as nonalcoholic fatty liver disease^35^, diabetes^36^ and atherosclerosis^37^. However, the role of autophagy in obesity-induced spermatogenesis deficiency still remains unknown.

In this study, we used HFD to induce obesity. Both activator and inhibitor of autophagy were employed in this study to explore the role of autophagy in HFD-induced spermatogenesis deficiency. Rapamycin (RAP), which inhibits the mammalian target of RAP (mTOR) signalling pathway by binding directly to the mTOR complex, and acts as an inducer of autophagy by activating the initiation stage. Chloroquine (CQ), which reverses autophagy by inhibiting lysosomal acidification, results in lysosome accumulation and autophagy blockade. Then, we investigated the role of autophagy in HFD-induced spermatogenesis deficiency by employing CQ to inhibit autophagy and RAP to activate autophagy in mice. The fertility of obese mice was also investigated after CQ and RAP treatment. 3-methyladenine (3-MA), based on its inhibitory effect on class III phosphatidylinositol-3 kinase (PI3K) activity, is another widely used autophagy inhibitor. Palmitic acid (PA) was used in the *in vitro* study to mimic the conditions *in vivo* as described previously^38^,^39^. To exclude the off-target effects of CQ and the possibility that the protection of CQ was secondary to the loss of body weight, both CQ and 3-MA were administered via intratesticular injection or were co-cultured with PA *in vivo* and *in vitro,* respectively. In addition, their effects on the viability and motility of mice sperm were explored in this study. Human semen samples were also collected to examine the potential effects of autophagy on the sperm quality of obese subfertile male patients.

## Results

### Autophagy is over activated in the testis of mice subjected to HFD

**Figure 1:**
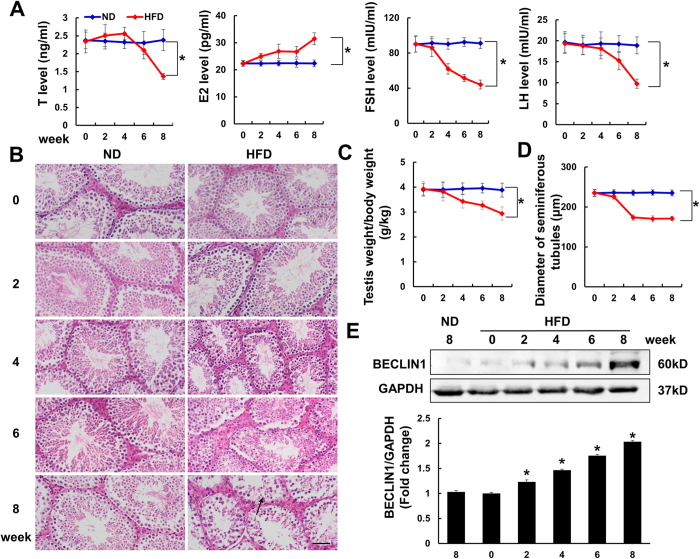
High-fat diet (HFD) induced male mice spermatogenesis impairment. (**A**) Serum hormone levels of testosterone (T), oestradiol (E2), follicle stimulating hormone (FSH) and luteinizing hormone (LH) (n = 6). ND is defined as normal diet. (**B**) Hematoxylin and eosin (HE) staining of testis of the indicated groups. Vacuoles in the testis are marked with arrow. Magnification: x200 Scale bar: 50 μm (n = 6). (**C**) Statistical results of testis weight/body weight of the indicated groups (n = 6). (**D**) Statistical analysis of the diameter of seminiferous tubules in four groups (n = 6). (**E**) Protein level of autophagy marker BECLIN1 in mice testis (n = 6). Full-length gels are presented in Supplementary Figure S4. Data are expressed as mean ± standard deviation (SD). *p < 0.05, compared with control group.

As shown in Fig. 1, the mice fed an HFD had impaired spermatogenesis, as manifested by abnormal serum sex hormone levels (Fig. 1A), decreased testis weight/body weight ratio (Fig. 1C) and pathological histological analysis (Fig. 1B), including atrophied seminiferous tubules (Fig. 1D) and an increased number of vacuoles. Meanwhile, after 8 weeks of the HFD, the mice became obese, and the serum cholesterol level was increased (see Supplementary Figure S1). The blood glucose was also detected. After 8 weeks of HFD feeding, the blood glucose was slightly increased. We also use the homeostasis model assessment of insulin resistance (HOMA-IR) to evaluate insulin resistance and found that there was no difference between the control group and HFD group (see Supplementary Figure S1). The autophagy-related protein BECLIN1 was detected to reflect the level of autophagy in the testis of mice subjected to HFD. As indicated, BECLIN1 was found to increase in the HFD group in a time-dependent manner (Fig. 1E and Supplementary Figure S4).

### Autophagy is involved in spermatogenesis deficiency induced by HFD

**Figure 2:**
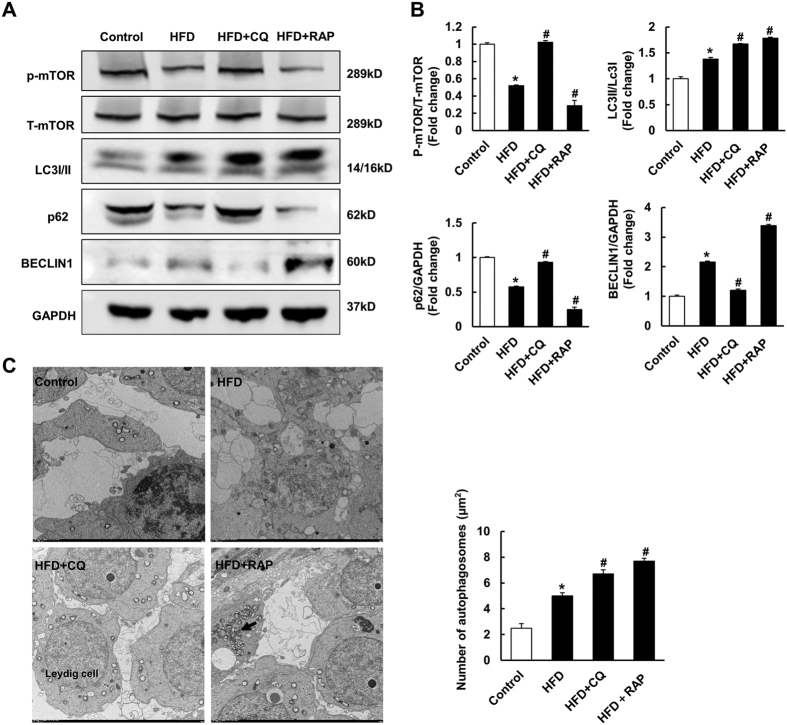
Intraperitoneal injection of chloroquine (CQ) and rapamycin (RAP) on testis autophagy. **(A**,**B**) The protein levels of autophagy related markers in mice testis in indicated groups (n = 6). Full-length gels are presented in Supplementary Figure S4. (**C**) The presence of autophagosomes (arrow) in Leydig cells of mice testis by transmission electron microscopy (TEM) examination of four groups (n = 6). Data are expressed as mean ± SD. *p < 0.05, compared with control group. ^#^*p* < 0.05, compared with HFD group.

**Figure 3:**
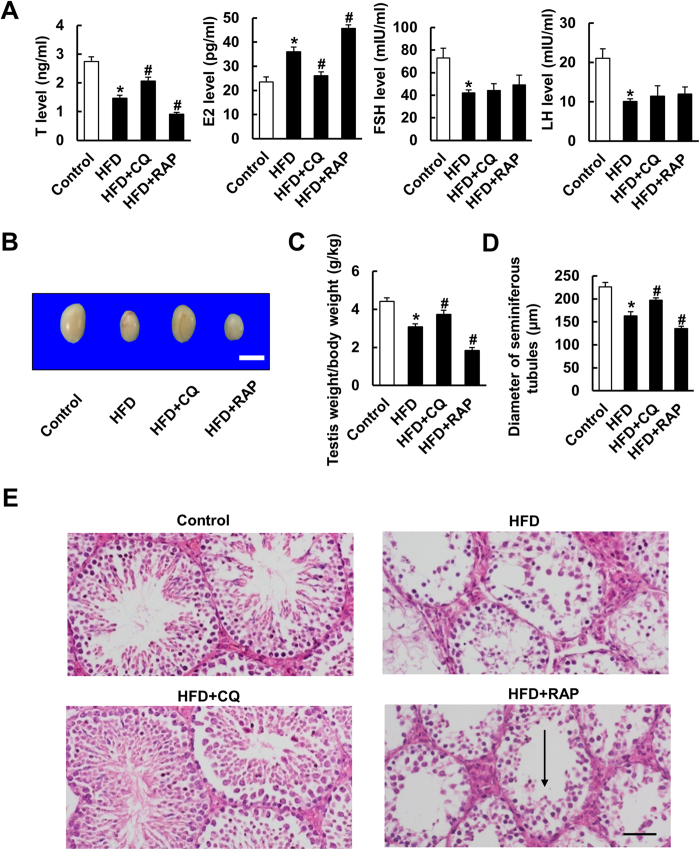
Suppressing autophagy by CQ ameliorated HFD-induced spermatogenesis deficiency. (**A**) Serum hormone levels of T, E2, FSH and LH of the four groups (n = 6). (**B**) Gross morphology of testis in the indicated groups Scale bar: 2 cm. (**C**) Statistical results of testis weight/body weight ratio of the indicated groups (n = 10). (**D**) Statistical analysis of the diameter of seminiferous tubules in four groups (n = 10). (**E**) HE staining of testis of the indicated groups (n = 10). Vacuoles in the testis were marked with arrow. x200 Scale bar: 50 μm. Data are expressed as mean ± SD. *p < 0.05, compared with control group. ^#^*p* < 0.05, compared with HFD group.

To investigate the role of autophagy in HFD-induced spermatogenesis impairment, CQ and RAP were injected intraperitoneally to inhibit autophagy and induce autophagy, respectively. As illustrated, autophagy was suppressed in mice with CQ injection, as indicated by decreased protein levels of BECLIN1 and increased LC3II/LC3I, p-mTOR and p62 levels. Conversely, the mice treated with RAP showed increased protein levels of BECLIN1 and LC3II/LC3I and decreased p-mTOR and p62 levels, which implied that autophagy was further promoted (Fig. 2A,B and Supplementary Figure S4). As CQ inhibits the degradation of autophagosomes, autophagosomes were increased in both the CQ and RAP treatment groups (Fig. 2C). Subsequently, spermatogenesis function was assessed. As shown in Fig. 3, CQ treatment attenuated HFD-induced spermatogenesis deficiency, as evidenced by increased size of the testis, an improved testis weight/body weight ratio, increased diameter of the seminiferous tubules, and improved serum hormone levels. Conversely, RAP treatment aggravated all these pathological changes. Taken together, these data demonstrate that autophagy is closely involved in HFD-induced spermatogenesis impairment and that inhibiting autophagy with CQ improved spermatogenesis deficiency induced by HFD.

### Intraperitoneal injection of CQ on sperm count, viability and motility

**Figure 4:**
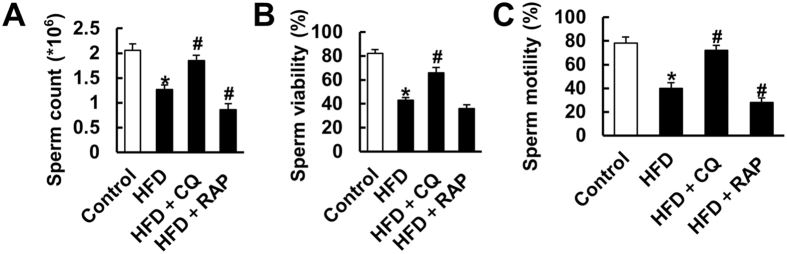
Inhibiting autophagy by CQ intraperitoneal injection increased sperm viability and motility in mice subjected to HFD. (**A**–**C**) Sperm parameters of mice in the indicated groups (n = 8). Data are expressed as mean ± SD. *p < 0.05, compared with control group. ^#^*p* < 0.05, compared with HFD group.

Inconsistent with a previous report showing that obese mice had no differences in total sperm count^40^, we found that an HFD led to a decrease in total sperm count. Moreover, HFD decreased the sperm viability and motility to 43% and 40%, respectively. Surprisingly, these pathological changes induced by HFD were attenuated after CQ treatment and, indeed, were even slightly potentiated by the addition of the activator of autophagy (Fig. 4).

### Intraperitoneal injection of CQ on fertility and adverse pregnancy outcomes

**Figure 5:**
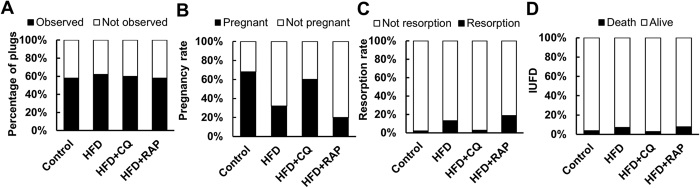
Inhibiting autophagy by CQ injection improved male mice subfertility induced by HFD. (**A**) Percentage of plugs of four groups. (**B**) Pregnancy rate of the indicated groups. (**C**) Embryo resorption rate of the indicated groups. (**D**) Intra-uterine foetal death (IUFD) of the four groups.

The fact that CQ could improve sperm quality in mice with HFD prompted us to explore the effect of CQ on the fertility of obese mice. Inconsistent with a previous study^40^, we found that the percentage of female mice with plugs did not change between the control and HFD groups (Fig. 5A). However, subsequent analysis showed that HFD decreased the pregnancy rate (68.0% vs 32.0%, *p* = 0.0003). As expected, the pregnancy rate increased to 60.0% (*p* = 0.005) after CQ treatment and decreased to 20.0% (*p* = 0.171) after RAP treatment (Fig. 5B). Embryo resorption was also observed in the pregnant mice (Fig. 5C). HFD led to an increase in the embryo resorption rate (2.1% vs 13.0%, *p* < 0.0001), and conversely, CQ treatment reduced the embryo resorption rate (2.9%, *p* = 0.003). Intra-uterine foetal death (IUFD) in the pregnant female mice mated with lean and obese male mice was counted, but no significant difference was found in the indicated groups (Fig. 5D).

### A single intratesticular injection of CQ and 3-MA improved the motility and viability of sperm *in vivo*

**Figure 6:**
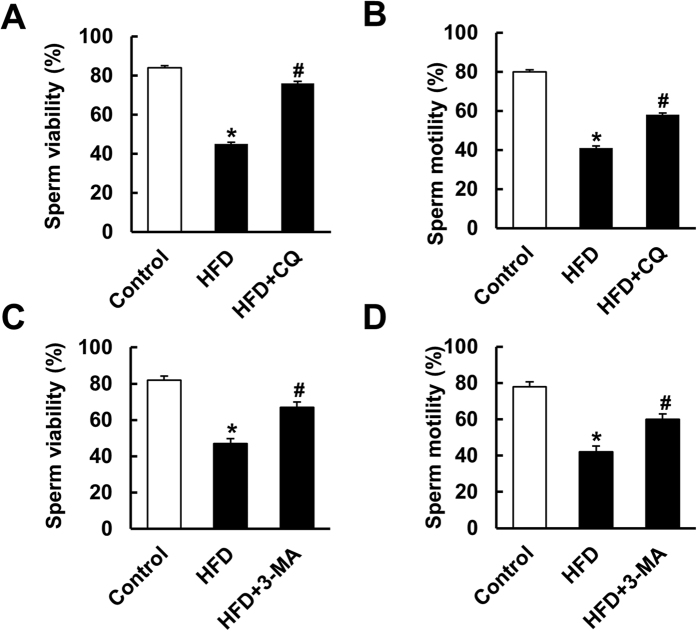
The effects of single intratesticular injection of CQ and 3-methyladenine (3-MA) on sperm viability and motility. (**A**,**B**) Sperm viability and motility after single intratesticular injection of CQ (n = 6). (**C**,**D**) Sperm viability and motility after single intratesticular injection of 3-MA (n = 6). Data are expressed as mean ± SD. *p < 0.05, compared with control group. ^#^*p* < 0.05, compared with HFD group.

To exclude the possibility that the protection of CQ against spermatogenesis impairment was secondary to the loss of body weight, CQ was administered via a single intratesticular injection. We decided the time point of intratesticular injection for the reason that after 4 weeks HFD feeding, the serum testosterone level and the testis weight/body weight began to decrease (Fig. 1). The morphological alteration of testis was also observed at 4 weeks after HFD (Fig. 1). To exclude the nonspecific effect of chloroquine and further confirm the role of inhibiting autophagy in spermatogenesis in HFD mice, mice were subjected to another autophagy inhibitor, 3-MA. As shown in Fig. 6, both CQ and 3-MA improved the motility and viability of sperm relative to that in the HFD group.

### CQ and 3-MA attenuated the reduction in the percentages of viable and motile spermatozoa caused by PA

**Figure 7:**
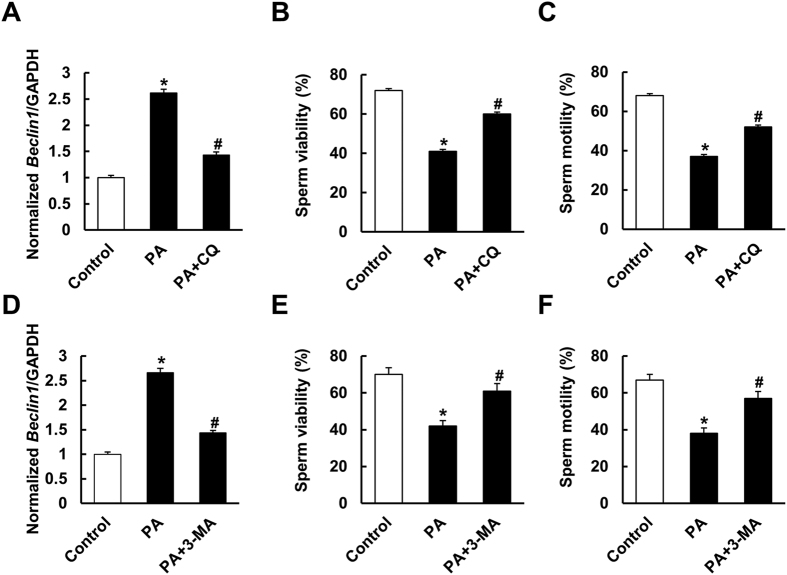
Suppressing autophagy improved palmitic acid (PA)-induced reduction of sperm viability and motility. (**A**) mRNA levels of *Beclin1* with CQ treatment (n = 6). (**B**,**C**) The effects of CQ on sperm viability and motility *in vivo* (n = 6). (**D**) mRNA levels of *Beclin1* with 3-MA treatment (n = 6). (**E**,**F**) The effects of 3-MA on sperm viability and motility *in vivo* (n = 6). Data are expressed as mean ± SD. *p < 0.05, compared with control group. ^#^*p* < 0.05, compared with PA group.

Considering the close relationship between elevated serum free fatty acid level and obesity^41^,^42^, and to further decipher the effect of autophagy inhibition, sperm were separated and co-cultured with PA *in vitro* to mimic the conditions *in vivo* as described previously^38^,^39^. Moreover, we found that the serum PA level and testis PA level were increased in the mice with HFD (see Supplementary Figure S2). Unpredictably, CQ or 3-MA impaired the viability and motility of sperm (see Supplementary Figure S3). However, both CQ and 3-MA clearly attenuated the reduction in viable and motile spermatozoa caused by PA (Fig. 7A–F).

### An increased level of BECLIN1 was observed in obese, subfertile male patients

**Figure 8:**
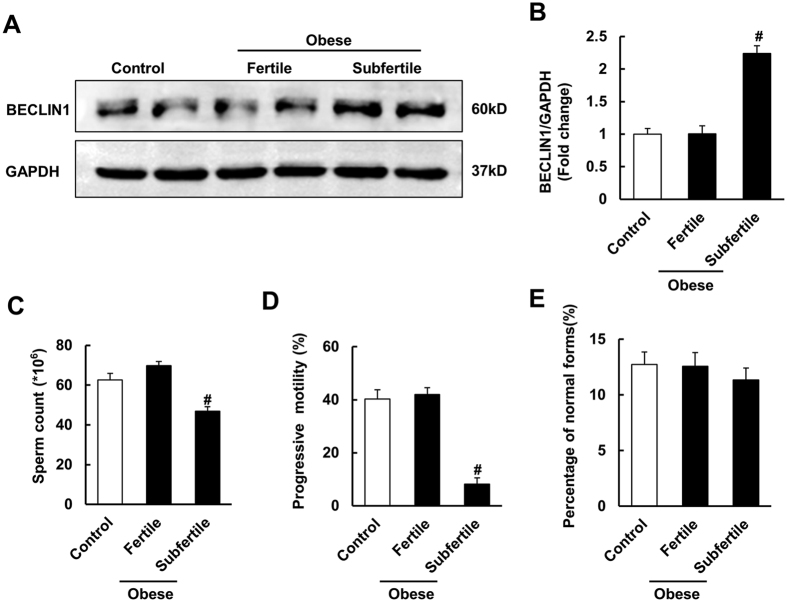
Autophagy was activated in obese and subfertile patients. (**A**,**B**) Protein level of BECLIN1 of human semen samples in the indicated groups. According to World Health Organization guidelines (REF: WHO 2010), the lower reference limits for semen parameters are: sperm concentration ≥15 × 10^6^ spermatozoa/ml, ≥32% progressively motile spermatozoa and ≥4% morphologically normal spermatozoa. Decreased semen quality in obese and subfertile patients reflects a condition when at least one of the parameters is under the lower reference limit. Control group: lean people with normal semen quality; obese and fertile group: obese people (BMI ≥ 30) with normal semen quality; obese and subfertile group: obese people with decreased semen quality. Full-length gels are presented in Supplementary Figure S4. (**C**–**E**) Semen parameters of human subjects in the indicated groups. Data are expressed as mean ± SD. ^#^*p* < 0.05, compared with control group.

According to World Health Organization guidelines (REF: WHO 2010), the lower reference limits for semen parameters are: sperm concentration ≥15 × 10^6^ spermatozoa/ml, ≥32% progressively motile spermatozoa and ≥4% morphologically normal spermatozoa. Decreased semen quality in obese and subfertile patients reflects a condition when at least one of the parameters is under the lower reference limit. BECLINI was found to be increased in obese patients with decreased semen quality, but no significant difference was found between normal individuals and obese and fertile patients, whose semen parameters were above the lower reference limits (see Supplementary Table S1 and Fig. 8A,B), suggesting an association with impaired sperm quality.

## Discussion

Autophagy has been implicated in the pathogenesis of different diseases such as cancer^43^,^44^, liver disease^45^,^46^,^47^, kidney disease^48^,^49^, neurodegenerative disease^50^,^51^ and cardiovascular disease^52^,^53^. Autophagy was also found to be associated with sperm survival^54^,^55^,^56^, and a recent study also showed that autophagy and apoptosis work synergistically to induce germ cell death during mouse spermatogenesis^30^. However, there are no related reports about the role of autophagy in HFD-induced male spermatogenesis impairment. The present study is the first to provide insight into the importance of autophagy during HFD-induced spermatogenesis deficiency and find that autophagy is over activated in HFD-induced spermatogenesis deficiency and CQ attenuates spermatogenesis impairment in HFD male mice. CQ also improves sperm count, viability and motility in mice subjected to HFD and decreases adverse pregnancy outcomes. In addition, both *in vivo* and *in vitro* studies demonstrated that CQ and 3-MA improved the reduction in sperm viability and motility induced by HFD and PA treatment, respectively. Moreover, the increased protein level of BECLIN1 in human semen samples, indicated that autophagy was activated in obese, subfertile male patients.

In this study, we showed that mice fed an HFD exhibited impaired spermatogenesis function in a time-dependent manner. Meanwhile, the serum steroid hormone levels were obviously changed, as demonstrated by an increased oestradiol (E2) level and decreased testosterone (T), follicle stimulating hormone (FSH) and luteinizing hormone (LH) levels. In addition, these changes were consistent with those of a previous study, which found that seminiferous tubule degeneration is associated with decreased FSH and LH levels^57^. The fertility of HFD male mice was also found to be damaged, which was in accordance with previous studies indicating that a hypercholesterolemic diet contributed to decreased semen quality and reproductive function in male rabbits^58^,^59^. Consistent with the previous finding that autophagy was increased in the testis of mice subjected to heat stress and exposed to formaldehyde^30^,^60^, we also found that autophagy was over activated in the testis of HFD mice, implying that autophagy may play a role in HFD-induced spermatogenesis deficiency. Previous study indicated that autophagy was activated in human spermatozoa and was involved in the regulation of cell survival and motility^18^. Consistent with this finding, the data in our study demonstrated that BECLIN1 was increased in semen samples from obese, subfertile male patients, implying a role of autophagy in human sperm production.

To the best of our knowledge, the primary role of autophagy is as a reparative and cellular cleaning process^61^,^62^. The role of autophagy in many other HFD-related diseases has been discussed in the past few decades. Autophagy markers were increased by HFD feeding in many reports^36^,^63^, which is consistent with our results, but there is still some evidence supporting a decreased autophagy level with HFD treatment^36^,^64^, and dual effects of autophagy in HFD mice were proposed. Recent studies have demonstrated that multiple forms of metabolic stress, including HFD and diabetes, provoke an increase in autophagic flux and that suppression of excessive autophagy could reduce or even reverse the maladaptive response^65^,^66^. Moreover, *Atg7* knockdown conferred protection against the heat-induced apoptosis of germ cells^30^. Consistent with these studies, our study demonstrated that CQ, by inhibiting autophagy, obviously attenuated HFD-induced spermatogenesis deficiency and infertility. Conversely, the mice treated with RAP displayed greater impairments in reproductive function. We also injected CQ and 3-MA directly into the testis and found that an improvement in spermatogenesis was undoubtedly produced by inhibiting autophagy. Taken together, these data demonstrated that inhibition of excessive autophagy could protect against HFD-induced spermatogenesis deficiency and subfertility.

The finding that CQ could improve impaired spermatogenesis does not support the notion that inhibiting autophagy is also a protective mechanism for spermatogenesis at basal condition. Conversely, mitophagy prevents paternal mitochondrial DNA transmission^67^. *Atg7* is required for acrosome biogenesis during spermatogenesis, and germ cell-specific *Atg7*-knockout mice display a phenotype similar to human globozoospermia^19^. It was also reported that the activation of autophagy is indispensable for the process of decidualization, which was confirmed in diet-induced obesity female mice^68^. Consistent with these facts, we also found that CQ impaired the viability and motility of sperm *in vitro* and *in vivo* at baseline, implying that autophagy is also indispensable for the physiological process of spermatogenesis. However, with regard to the role of autophagy in the pathological process, the results differ. Zhang *et al*. reported that autophagy was not a protective mechanism against heat-induced apoptosis^30^. Our data also demonstrated that CQ could improve the quality of sperm in mice fed an HFD by inhibiting autophagy. These discrepant results possibly reflect the different roles of autophagy in physiological and pathological processes. Indeed, the dual nature of autophagy is a recurring theme in other organ systems and disease states^31^. Therefore, further studies are required to elucidate the dual roles of autophagy in the production of sperm.

There are several types of cells in testis, including Leydig cells, Sertoli cells and spermatogenic cells. The role of autophagy in these cells during HFD-induced impaired spermatogenesis is still unknown. The finding in our study that CQ or 3-MA could attenuate PA-induced sperm injury imply that CQ or 3-MA may directly target on the sperm. Consistent with a previous study^69^, we also observe the autophagosomes in the Leydig cells, and the decreased T level induced by HFD was restored after CQ treatment, indicating that Leydig cells may mediate CQ-induced protection. The results that CQ or RAP can not affect the level of LH and FSH in the mice with HFD, implying that pituitary gland was not involved in the alteration of hormones caused by the treatment of CQ or RAP.

Despite the results that autophagy was over activated in spermatogenesis when males were exposed to an HFD, we do not yet know which molecules induced such activation. Indeed, an HFD induced the decreased phosphorylation of adenosine monophosphate-activated protein kinase (AMPKα)^39^ and thus inhibited the activation of mTOR, which is an agonist of autophagy^70^,^71^. Previous reports also demonstrated that autophagy is activated in response to different forms of metabolic stress, including nutrient deprivation, growth factor depletion, and hypoxia^31^,^32^. Whether these metabolic stresses play roles in HFD-induced spermatogenesis impairment remains unknown. Genetic approaches to inhibit autophagy will yield more conclusive information about the biologic roles of autophagy in spermatogenesis and HFD-induced spermatogenesis deficiency. In addition, our study does not decipher whether HFD-induced autophagy occurs in response to the inciting stress, is a secondary response, promotes or antagonizes disease, or exists as an epiphenomenon.

In conclusion, we have confirmed that autophagy is over activated in HFD-induced spermatogenesis deficiency and found that inhibiting the autophagic process ameliorates HFD-induced spermatogenesis impairment *in vitro* and *in vivo*. Our study also indicate that autophagy is increased in semen samples from obese, subfertile male patients. Taken together, our finding provides a new therapeutic target for infertility induced by obesity, and further investigations are required to clarify the specific molecular mechanism and signalling pathway that are involved in the activation of autophagy, especially in human subjects.

## Methods

### Reagents

CQ (100421-200401) was purchased from the National Institutes for Food and Drug Control (Beijing, China), and RAP (R-5000) was purchased from LC Laboratories (Woburn, MA, USA). 3-MA (M9281) was purchased from Sigma (St. Louis, MO, USA). The rabbit anti-SQSTM1/p62 monoclonal antibody (ab109012) for western blot was purchased from Abcam (Cambridge, UK). The rabbit anti-phospho-mTOR antibody (#2974), rabbit anti-mTOR antibody (#2983), rabbit anti-BECLIN1 antibody (#3495) and rabbit anti-LC3II/I antibody (#12741) for western blot were purchased from Cell Signaling Technology (Beverly, MA, USA). The rabbit anti-GAPDH antibody (sc-25778) for western blot was purchased from Santa Cruz Biotechnology (Dallas, TX, USA). The secondary antibody was purchased from LI-COR Biosciences. Oestradiol (E2, E-EL-0065c), testosterone (T, E-EL-0072c), follicle stimulating hormone (FSH, E-EL-M0511c), and luteinizing hormone (LH, E-EL-M0057c) detection kits were purchased from Elabscience Biotechnology Co., Ltd. (Wuhan, China). PA (P9767) was purchased from Sigma (St. Louis, MO, USA). All the other chemicals used in our study were of analytical grade.

### Animal care and treatment

All the animal experiments in our study were performed in accordance with the Guidelines for the Care and Use of Laboratory Animals published by the United States National Institutes of Health (NIH Publication, revised 2011) and the Guidelines for the Care and Use of Laboratory Animals of the Chinese Animal Welfare Committee and were approved by the Animal Use Committees of Renmin Hospital of Wuhan University. Male C57BL mice (Permit number: 42000600004159, body weight: 20–22 g) were obtained from Hubei Provincial Center for Disease Control and Prevention (Wuhan, China). The mice were housed under a 12 h light: 12 h dark cycle in a temperature (20–25 °C) and humidity (50 ± 5%)-controlled environment with free access to food and water. Mice (n = 40) were randomly divided into four groups, including a control group (Normal diet + 0.1% DMSO, n = 10), an HFD group (HFD + 0.1% DMSO, n = 10), an HFD + CQ group (n = 10) and an HFD + RAP (n = 10) group. All the mice were fed ad libitum either a standard chow or a HFD. Subsequently, the mice in the HFD + CQ or HFD + RAP groups were intraperitoneally injected with CQ (10 mg/ kg)^72^ or RAP (1 mg/ kg)^73^ once per day for 8 weeks^74^,^75^, and the mice in the control group were given the same volume of vehicle. CQ was dissolved in sterile saline, and RAP was dissolved in 0.1% DMSO. After that, the mice were anaesthetized with an intraperitoneal injection of 3% sodium pentobarbital, and blood samples were obtained from the angular veins. Then, mice were euthanized with an intraperitoneal injection excessive sodium pentobarbital, the testes were collected for further experiments and the testis weight/body weight ratio was calculated. To further confirm the effect of autophagy inhibition on spermatogenesis in HFD male mice, a single intratesticular injection was performed. After mice had received the HFD for 4 weeks, 40 μl of the autophagy inhibitor CQ (300 μM) or 3-MA (10 mM, dissolved in sterile saline)^19^ was injected under the tunica vaginalis of the testes with a 50 μl micro-injector at a depth of 5 mm under the tunica albuginea as described previously^76^. After injection, the mice were fostered for another 4 weeks. Spermatozoa were taken from the epididymis to examine their viability and motility.

### Determination of serum hormone levels

Blood samples were collected from angular veins. After centrifuging the samples at 1409 *g* and 4 °C for 15 minutes, the supernatant sera were obtained and stored at −80 °C until analysis. The levels of T, E2, FSH and LH in the serum were measured using commercially available ELISA kits according to the manufacturer’s instructions. Briefly, ninety-six-well plates were coated with the primary antibody at 37 °C for 2 h. After being blocked with 5% milk, the serum were added into the plates. The plates were incubated at 37 °C for 2 h. After that, the plates were incubated with peroxidase-conjugated second antibodies at 37 °C for 1 h. After that, the optical density values were detected by a microplate reader (Biotek, Vermont, USA).

### Histological analysis

The sections of testis tissue were routinely fixed in Bouin’s solution, dehydrated, and embedded in paraffin. Testis tissue sections were stained with haematoxylin and eosin (H&E) to examine morphology. Sections (5 μm) were viewed under light microscopy (Nikon E100), and photomicrographs were captured with the Photo Imaging System (Canon 600D). The diameter of seminiferous tubules was determined using Image-Pro Plus 6.0. In each group, 100 seminiferous tubules (5 fields per mouse, 2 random seminiferous tubules per field) were measured in 10 mice, and the mean seminiferous tubule diameter was determined.

### Protein extraction and western blot assay

RIPA buffer (720 μl radioimmunoprecipitation buffer, 100 mmol/l PMSF, 100 μl cocktail, 100 μl Phos-stop, 20 mmol/l NaF and 100 mmol/l Na_3_VO_4_ in 1 ml) was used to extract the proteins from testis tissue of mice and sperm samples of mice and humans^77^. The protein concentrations were detected with a BCA Protein Assay Kit (Pierce, Rockford, IL, USA). Proteins (20 μg) were separated on SDS-PAGE gels (LC3I/II: 15%; mTOR and p-mTOR: 6%; Others: 10%) for 2 h (75 V 30 min; 100 V 90 min) at room temperature and then transferred to a PVDF membrane (IPVH00010, Millipore, Billerica, MA, USA) at 4 °C for different times (200 mA; LC3I/II: 70 min; mTOR and p-mTOR: 120 min; Others: 90 min). After being blocked with 5% non-fat milk, the membranes were incubated overnight at 4 °C with the following primary antibodies: rabbit anti-GAPDH antibody (1:5000), rabbit anti-BECLIN1 antibody (1:1000), rabbit anti-SQSTM/p62 monoclonal antibody (1:10000), rabbit anti-LC3II/I antibody (1:1000), rabbit anti-phospho-mTOR antibody (1:1000) and rabbit anti-mTOR antibody (1:1000). After incubation with the secondary antibody (1:5000), the membranes were scanned using a two-colour infrared imaging system (Odyssey, LI-COR, Lincoln, NE, USA). Densitometry analysis was performed by Odyssey as described previously^78^, and the results were normalized to GAPDH.

### RNA isolation and qPCR analysis of mouse semen samples

Sperm samples of mice were homogenized in TRIzol (Invitrogen, Massachusetts, USA) on ice, and total mRNA was extracted. mRNA was reverse transcribed into cDNA using the Transcriptor First Strand cDNA Synthesis Kit (04896866001, Roche, USA) according to the manufacturer’s instructions. All experiments were performed in triplicate, and quantitative RT-PCR analysis was carried out using the Light Cycler 480 SYBR Green 1 Master Mix (04707516001, Roche, USA). The primer sequences for *Beclin1* were: forward: 5′-ATCCTGGACCGTGTCACCATCCAGG-3′; reverse: 5′-GTTGAGCTGAGTGTCCAGCTGG-3′. The primer sequences for *Gapdh* were: forward: 5′-GTTGTCTCCTGCGACTTCA-3′; reverse: 5′-GGTGGTCCAGGGTTTCTTA-3′. The mRNA levels were analysed using the 2^−ΔΔCt^ method and normalized to *Gapdh*. The qPCR analysis was performed as described in a previous report^79^.

### Transmission electron microscopy (TEM)

Testis tissue was fixed with 2.5% glutaraldehyde, and then these tissues were post-fixed in 1% OsO_4_ for 2 h. After rehydration, these tissues were embedded in epoxy resin. Ultrathin sections (80 nm) were collected on formvar-coated copper grids. Subsequently, these grids were counter-stained with uranyl acetate and lead citrate and examined on an EM2010FEF-Ω transmission electron microscope (JEOL, Tokyo, Japan). Autophagosomes were detected as described in a previous study^80^ and identified by the presence of a double-membrane vesicle that contained cytosol and/or organelles and looked morphologically intact.

### Semen analysis in mice

The epididymis was carefully dissected away from the fat. The separated epididymides were immediately placed into Ringer’s solution and dissected to determine the count, viability and motility of the sperm as described in a previous study^40^. The sperm were gradually pressed out from the epididymis and collected in an Eppendorf tube. The sperm number was counted with a haemocytometer independently for three times.

Eosin-nigrosin staining solution (1% eosin and 10% nigrosin) was used to detect sperm viability. Semen samples were first incubated with the eosin-nigrosin staining solution and smeared on a microscope slide. The spermatozoa were observed under a light microscope at 100x magnification. Live spermatozoa is not stained, while dead spermatozoa with disintegrating cell membranes take up the stain^81^. Sperm motility was tested with computer-assisted sperm analysis (CASA). Sperm were incubated in Ringer’s solution at room temperature for 30 minutes and placed into CASA assay chambers (Hamilton Thorne Research, Beverly, MA, USA). Sperm tracks (1.5 s, 30 frames) were captured at 60 Hz and were analysed with HTM-IVOS Sperm Analyzer software (version 12.2 L; Hamilton Thorne)^82^. Five hundred sperm cells were randomly selected and evaluated to obtain the sperm viability and sperm motility; at least three replications were completed for each sample.

### Assessment of the fertility of male mice

At the end of the 8-week HFD, the mice receiving different treatments were used in a breeding assay. Each male mouse (n = 5) was caged with two females for five days; consequently, a total of 50 females were involved in the study, and their vaginal plugs were checked every morning. The day on which the vaginal plug observed was defined as day 0.5 of gestation, and on day 14.5 of gestation, female mice were euthanized to count the number of dead foetuses and resorptions.

### *In vitro* study

Mouse sperm samples obtained from the cauda epididymis were diluted to attain a concentration of 5 × 10^6^ spermatozoa/ml and incubated in G-IVF (Vitrolife, Sweden) medium, fatty medium (1 mM PA)^83^ or fatty medium with CQ (100 μM) and 3-MA (10 mM)^84^. Incubations were performed under a humidified atmosphere of 5% CO_2_/95% O_2_ and 37 °C for 5 h, and sperm viability and motility were assessed as described above. Three independent experiments were carried out in the *in vitro* study.

### Human studies

The human studies conformed to the Declaration of Helsinki and were approved by the Human Research Ethics Committees of Renmin Hospital of Wuhan University in Wuhan, China. All the participants in this study were interviewed after signing an informed consent. Semen samples were obtained from subjects who attended the infertility clinic by masturbation after 3–7 days of sexual abstinence. The interviewers collected the data of all participants, including their ethnicity, age, height, body weight, medical history, and lifestyle through a structured questionnaire. The subjects were ethnic Han Chinese from Hubei province and its nearby regions. They were men who visited the Reproductive Medical Center of Renmin Hospital of Wuhan University for infertility treatment from June 2014 to December 2014. All subjects were age-matched, and men who were unhealthy with other causes of defective spermatogenesi^85^, including infection, varicocele, obstruction of the vas deferens, chromosomal abnormalities or smokers, were excluded. Finally, all semen samples were divided into three groups, including the control group (n = 85), obese and fertile group (n = 65), and obese and subfertile group (n = 79). The semen samples in the control group were from individuals with normal body mass index (BMI) and normal semen parameters as described above. The semen samples in the obese and fertile group were from men whose BMIs were more than 30 and had normal semen quality, while the semen samples in the obese and subfertile group were from those whose BMIs were more than 30 and had decreased semen quality. The semen samples were liquefied for 15–30 minutes at 37 °C, and subsequently the semen parameters were tested in accordance with the World Health Organization guidelines. The residual semen samples were used for protein extraction and subsequent western blotting analysis.

### Data analysis

Group data are reported as the mean ± standard deviation (SD). All statistical tests were performed with SPSS 19.0. All the data in the present study were normally distributed (*p* < 0.05), as determined by the Kolmogorov-Smirnov’s test. Two-group comparisons were analysed with an unpaired Student’s t-test after confirming equality of variance with the F test. Multiple-group comparisons were performed using one-way ANOVA followed by Tukey’s post hoc test^70^ when ANOVA analysis indicated a significant value of F and no variance in homogeneity; otherwise, Tamhane’s T2 post hoc test was used. The percentage of plugs, the pregnancy rate, the resorption rate and the IUFD between different groups were compared using a chi-square test. *P*-values less than 0.05 were considered significant.

## Additional Information

**How to cite this article**: Mu, Y. *et al*. Diet-induced obesity impairs spermatogenesis: a potential role for autophagy. *Sci. Rep.* **7**, 43475; doi: 10.1038/srep43475 (2017).

**Publisher's note:** Springer Nature remains neutral with regard to jurisdictional claims in published maps and institutional affiliations.

## Supplementary Material

Supplementary Information
