# Supplementary material for: Diet-induced obesity impairs spermatogenesis: a potential role for autophagy
Source: Sci Rep. 2017 Mar 9;7:43475. doi: 10.1038/srep43475 (PMC5343591; doi:10.1038/srep43475)
Supplement: Supplementary Information [file srep43475-s1.pdf]

Supplementary Information

Diet-induced obesity impairs spermatogenesis: a potential role for autophagy

Yang Mu[1], Wen-jie Yan[1], Tai-lang Yin[1], Yan Zhang[2], Jie Li[1], Jing Yang[1*]

[1]Reproductive Medicine Center, Renmin Hospital of Wuhan University, Wuhan 430060, China

[2]Department of Obstetrics and Gynecology, Renmin Hospital of Wuhan University, Wuhan 430060, China

[*]Corresponding author

Dr. J Yang. Reproductive Medicine Center, Renmin Hospital of Wuhan University, Wuhan University at Jiefang Road 238, Wuhan 430060, China. Tel.: +86 13507182023; Fax: +86 27 88080974. E-mail address: dryangqing@hotmail.com.

Table S1

Age, height, weight, body mass index (BMI) and blood glucose of study subjects

Parameter	Control （n=85）	Obese and fertile (n=65)	Obese and subfertile (n=79)
Age	32.84±1.61	32.87±1.23	33.50±1.37
Height (cm)	173.97±5.05	171.85±4.57	172.13±4.84
Weight (kg)	76.71±4.58	94.54±6.34*	95.00±8.01*
BMI (kg/m^2)	25.34±0.89	31.87±1.87*	32.06±2.22*
Blood glucose (mmol/L)	4.95±0.27	5.05±0.33	5.07±0.37

* p<0.05, compared with control group.

Table S2

Macronutrient composition of diets for mice

	Control		HFD	
	g%	kJ%	g%	kJ%
Protein	20	19	20	14
Carbohydrate	76	72	45	31
Saturated fat	4	9	35	55
kJ/g	17.5		24.1	

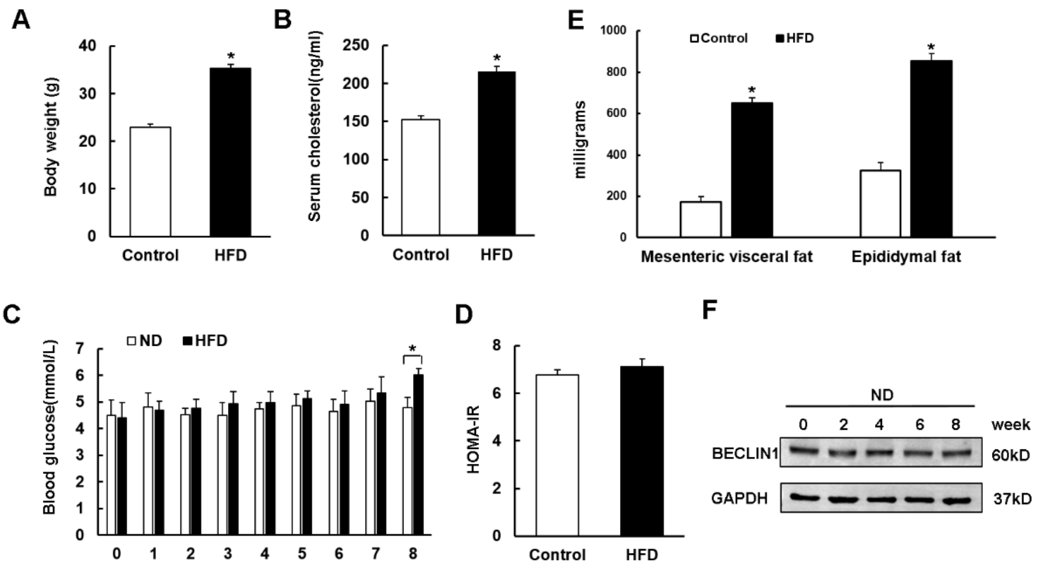

Figure S1. Metabolic changes of high-fat diet (HFD) mice. (A) Body weight of control and HFD groups (n=10). (B) Serum cholesterol levels of control and HFD groups (n=6). (C) Blood glucose levels of control and HFD groups (n=6). (D) HOMA-IR of the control and the HFD groups (n=6). (E) Mesenteric visceral fat weight and epididymal fat weight of control and HFD groups (n=6). (F) Protein level of BECLIN1 of the normal diet group in corresponding ages (n=6).Data are expressed as mean ± SD. $^{*}p<0.05$, compared with control group.

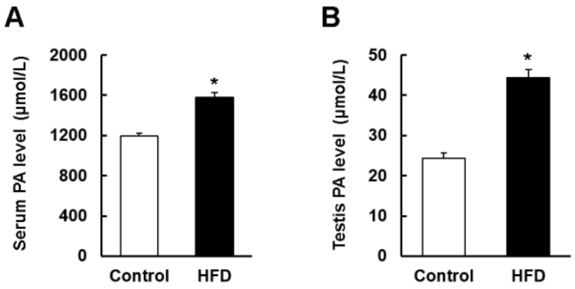

Figure S2. Serum palmitic acid (PA) level and testis PA level of the control and HFD groups. (A) Serum PA level of the control and HFD group (n=6). (B) Testis PA level of the control and HFD group (n=6). $^{*}p<0.05$, compared with control group.

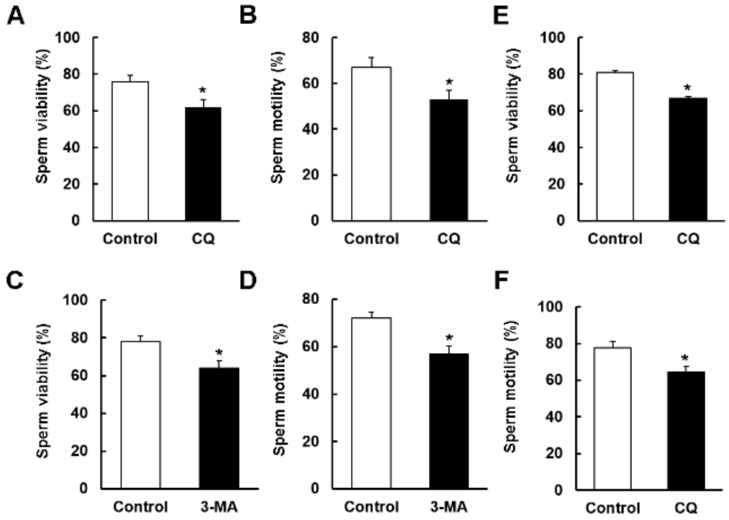

Figure S3. CQ or 3-MA decreased sperm viability and motility *in vitro* and *in vivo* at baseline. (A) Sperm viability of control and CQ groups *in vitro* (n=6). (B) Sperm motility of control and CQ groups *in vitro* (n=6). (C) Sperm viability of control and 3-MA groups *in vitro* (n=6). (D) Sperm motility of control and 3-MA groups *in vitro* (n=6). (E) Sperm viability of control and CQ groups *in vivo* (n=6). (B) Sperm motility of control and CQ groups *in vivo* (n=6). Data are expressed as mean ± SD. $^{*}p<0.05$, compared with control group.

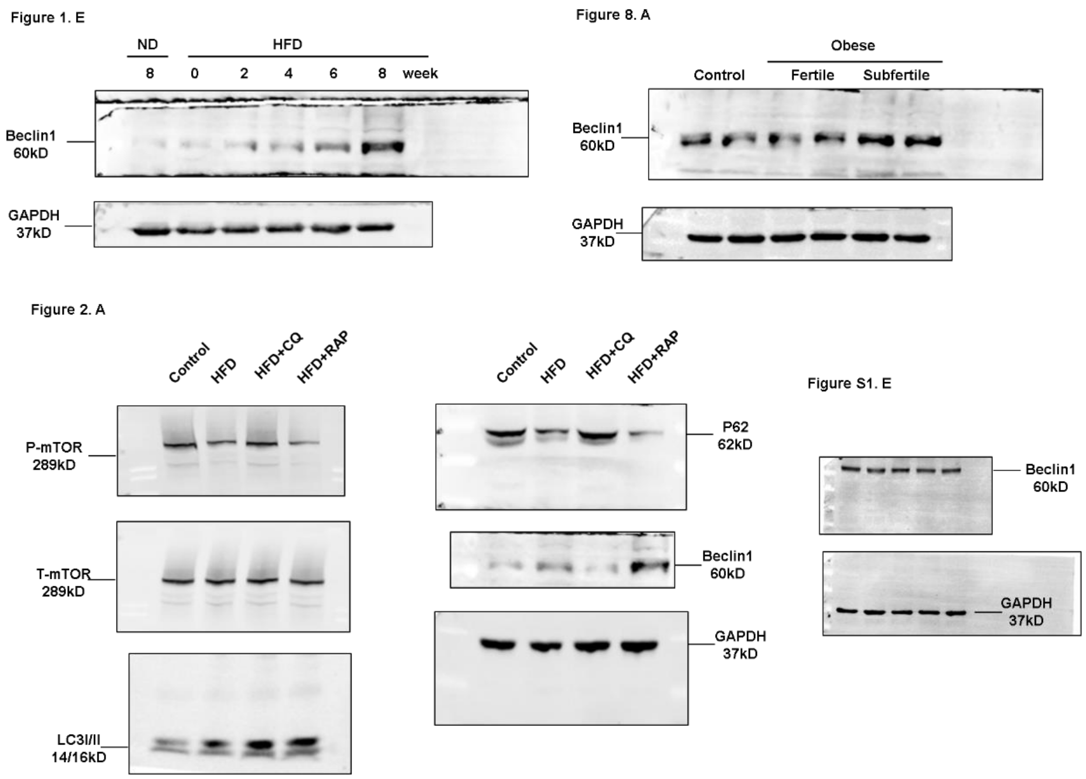

Figure S4. Full gel scans relating to indicated figures.
